# Developing a Prototype Home‐Based Toothbrushing Support Tool for Families in Scotland: A Mixed‐Methods Study With Modified Delphi Survey and Semi‐Structured Interviews

**DOI:** 10.1111/cdoe.13031

**Published:** 2025-02-12

**Authors:** Emma Fletcher, Andrea Sherriff, Denise Duijster, Maddelon de Jong‐Lenters, Al Ross

**Affiliations:** ^1^ Community Oral Health Group, University of Glasgow Dental School, School of Medicine, Dentistry & Nursing, College of Medical, Veterinary & Life Sciences University of Glasgow Glasgow UK; ^2^ Department of Oral Public Health, Academic Center for Dentistry Amsterdam University of Amsterdam and VU University Amsterdam the Netherlands; ^3^ Department of Pediatric Dentistry, Academic Center for Dentistry Amsterdam University of Amsterdam and VU University Amsterdam the Netherlands; ^4^ School of Health, Science and Wellbeing Staffordshire University Stoke‐on‐Trent Staffordshire UK

**Keywords:** child oral health, qualitative research, supervised toothbrushing

## Abstract

**Background:**

Scotland's National Oral Health Programme for Children, Childsmile, provides targeted home toothbrushing support for families of young children (0–3 years) in the home setting. The study describes the adaptation of an existing dental practice‐based intervention from the Netherlands using pictorial cards (*Uitblinkers*) for use in the programme. The aims were to modify Uitblinkers for the setting and context in Scotland by: (1) identifying the barriers that parents/carers in need of extra support face in implementing supervised toothbrushing; (2) explore consensus about behaviour change techniques that are appropriate and valid to address these; and (3) making recommendations for the design of a co‐produced home‐support tool and identifying facilitators for implementation in practice.

**Methods:**

A modified Delphi study was carried out consisting of two survey rounds with a purposively recruited expert panel (*n* = 21) to develop consensus on home toothbrushing barriers (aim 1), behaviour change techniques (aim 2) and considerations for implementation (aim 3). Proposition statements for the Delphi were derived from literature, discussions with project advisors and from *Uitblinkers*, an existing behaviour change intervention for parents developed by the Academic Centre for Dentistry Amsterdam (ACTA) and delivered in dental practice. Then 12 in‐depth, semi‐structured interviews were conducted with Dental Health Support Workers in Scotland (delivering the home support toothbrushing intervention) to gather the views on the proposed toothbrushing barriers, behaviour change techniques and considerations for implementation (aim 1 to 3). Delphi results are presented descriptively in terms of percentage agreement and priority ratings. Interview transcripts were analysed using Template Analysis.

**Results:**

From the Delphi study, a final set of 11 overlapping child, parent and environmental/social toothbrushing barriers was agreed upon (aim 1), to be addressed through a tool based on applied Motivational Interviewing, and a combination of Operant Conditioning, Stimulus Control and Goal‐Setting techniques (aim 2). Experts supported the tool as realistic for delivery in the home setting, provided staff were trained. A physical ‘paper’ tool was preferred to a proposed electronic version (aim 3). Themes from interviews were: (1) the barriers present an exhaustive set and are valid from staff experience with families; (2) Motivational interviewing is appropriate and fits with usual practice; (3) the included behaviour change techniques are workable; (4) the tool is generally feasible within the operation of Childsmile home visits; (5) the tool is not less applicable for children with additional support needs.

**Conclusions:**

A card‐based conversational intervention to provide targeted home toothbrushing support for families of young children (0–3 years) in the home setting in Scotland, drawing from a template from the Netherlands, has been deemed worthy of further testing based on expert consensus and staff views on barriers faced, appropriate behaviour change techniques to address these and the design of a physical tool.

## 1 Introduction

It was reported in the early 2000s that the oral health of Scottish children was poor with 55% of 5‐year olds having obvious decay experience [1]. In addition, a larger proportion of children living in the most deprived areas experienced dental caries compared with those living in less deprived areas. In response, in 2005 the Scottish Government published “An action plan for improving oral health and modernising dental services in Scotland” [2]. This led to the development of the Childsmile programme, the main aims of which are to improve children's oral health and reduce inequalities in both oral health and access to dental services [3]. Childsmile is comprised of both universal and targeted elements which are monitored by ongoing and extensive evaluation methods [4]. There has since been a reduction in the prevalence of dental caries, with 27% of Scottish 5‐year olds having obvious decay experience in 2022 [5]. However, despite overall improvements, inequalities persist with 42% of 5‐year‐old children living in the most deprived areas experiencing decay compared to 14% in the least deprived areas.

A key component of the Childsmile programme is the universal supervised fluoride toothbrushing programme in all nurseries in Scotland and this intervention is associated with reduced odds of caries experience [6]. Targeted support in the home setting is delivered by Dental Health Support Workers (DHSWs) following a referral from a Health Visitor who has identified that the family may require additional support with caring for their child's oral health [7]. One role of DHSWs is to support families to improve early oral health behaviours at an early age before children are participating in the supervised toothbrushing programme at nursery at 3 years old. However, there may be barriers which limit families' abilities to the implement effective toothbrushing and these must be addressed in order to allow a toothbrushing routine to be established.

The *Uitblinkers* (translation “brilliant stars”) intervention, which was developed by the Academic Centre for Dentistry Amsterdam (ACTA) in 2017, is a behaviour change intervention for parents to promote twice daily toothbrushing in children aged 2–10 years. The intervention is delivered by dental care professionals in the practice setting and addresses parental barriers to toothbrushing by using principles from social learning theory [8]. It involves conversational techniques aided by the use of pictorial cards whereby parents choose barriers to toothbrushing that they identify with (example: ‘Toothbrushing is challenging when my child is too tired’, ‘Toothbrushing is challenging when I am stressed or pre‐occupied’ [See Data S1]). Subsequently, possible strategies using the principles of Stimulus Control, Operant Conditioning [9] and positive parenting from social learning theory [10] are outlined so that staff can work with families in addressing the specific barrier they have identified with.

Stimulus Control and Operant Conditioning are proven concepts from behavioural psychology that state that new behaviours or changes in behaviours are learned through repeated associations between stimuli and response. Stimulus Control refers to controlling the environmental context within which a behaviour occurs [11]. Parenting practices related to Stimulus Control are focused on creating conditions at home that promote desired child behaviour through structuring time and space (e.g. performing tasks in a fixed order and at a fixed place), introducing rules and routines and setting clear boundaries. Operant Conditioning can be defined as a learning process by which a person's behaviour changes in response to the consequences of that behaviour [9, 12]. Parenting skills related to Operant Conditioning focus on positive (intermittent) reinforcement of desired behaviours of a child, for example, through praise and reward. A negative stimulus (e.g. ignoring child resistance, or a form of admonishment) in the case of undesired behaviour can be used to decrease the likelihood of repetition. In the *Uitblinkers* intervention these parenting principles are used to help parents address barriers to toothbrushing.

The aims of this study were to modify Uitblinkers for the setting in Scotland by: (1) identifying the barriers that parents/carers in need of extra support face in implementing supervised toothbrushing; (2) explore consensus about behaviour change techniques that are appropriate and valid to address these; and (3) making recommendations for design of a co‐produced home‐support tool and identifying facilitators for implementation in practice.

## 2 Methods

This was a mixed‐methods study, containing a modified Delphi survey with an expert panel, followed by semi‐structured interviews with support workers who are users of the proposed intervention.

### 2.1 Modified Delphi Methodology

Part 1 included a Delphi survey. The Delphi technique gathers and summarises collated expert opinion and is widely applied in health research [13], most often when trial evidence is lacking and/or guidance is required. ‘Modified’ refers to a flexibility in approach in terms of numbers or characteristics of participants and closed or open question sets [14]. The process involves presenting ideas or propositions and assessing consensus via agreement, priority ratings or similar judgements. It is iterative whereby participants are fed back results and thereby allowed to reassess and/or comment on the group consensus [15, 16] and responses are generally gathered and fed back anonymously [17]. A four‐round modified Delphi technique was employed, with rounds 1 and 2 gaining initial then iterated views on toothbrushing barriers and rounds 3 and 4 on behaviour change techniques and implementation issues.

### 2.2 Identifying Propositions for the Delphi Study

Propositions for consensus testing were generated from a range of sources. First, as is conventional for generating information for a Delphi study [18], a brief review of the existing literature was carried out to identify published barriers to parental supervised toothbrushing. The search strategy was adapted from a recent systematic review looking at home toothbrushing interventions for young children [19]. Databases used were PubMed, MEDLINE, Embase and Web of Science using the search terms ‘toothbrushing’, ‘tooth decay’, ‘children’ and ‘parent/carer’. The search was carried out between June to July 2022. Boolean operators (AND, OR, proximity) were used to create and refine the search. Inclusion criteria were: reporting of identified barriers to parent/carer home supervised toothbrushing for children; and availability in English language. Studies were excluded if: there was no parental involvement; the setting was not in the home (e.g. school or nursery). The reference lists of identified articles were also checked by hand for relevant studies. The process identified 18 relevant papers from which toothbrushing barriers were extracted (See Data S2). Proposed barriers also drew from the original nine barriers contained in the *Uitblinkers* intervention, and feedback following a preliminary workshop with Scottish dental teams where these were presented for discussion.

All identified barriers across these sources were collated and then mapped against categories from the Theoretical Domains Framework (TDF); [20]. The TDF was originally created for implementation research to identify influences on health professional behaviour in relation to implementation of evidence‐based recommendations [21]. It is a determinant framework for ensuring coverage of potential influences on health behaviours.

Proposed techniques were drawn from the Uitblinkers model and tool and covered Motivational Interviewing together with Stimulus Control, Operant Conditioning and Goal Setting. The final list of proposed barriers and techniques for consensus testing was reviewed by the research team alongside a home toothbrushing advisory committee consisting of: DHSWs, an associate professor in Dental Public Health and paediatric dentist from ACTA who created the *Uitblinkers* intervention, and a Psychologist and Professor of Dental Public Health both of whom are involved in the development of recent interventions to improve home toothbrushing in children in deprived areas in England.

### 2.3 Qualitative Interview Methodology

Part 2 of the study involved in‐depth, semi‐structured interviews with DHSWs, analysed via Template Analysis (TA). The interview guide was developed to answer the research aims. Participants were presented with the outline of the intervention and asked questions on: barriers to family toothbrushing; appropriate behaviour change techniques to address these; aspects of design of the tool and practical implementation. Implementation questions were theory‐based, adapted from the Consolidated Framework for Implementation Research (CFIR) [22].

### 2.4 Procedures

Ethical approval for both studies was obtained from the University of Glasgow Ethics Committee (Project number MVLS200150076).

### 2.5 Delphi Procedures

Expert researchers and clinicians were identified from a review of the literature on child toothbrushing barriers and purposively selected [23] as being able to inform the aims of the study and constituting a heterogenous group [24]. Rounds 1 and 2 (toothbrushing barriers) involved those having research or practical experience in the area of child toothbrushing in the home setting and Rounds 3 and 4 (behaviour change and implementation) widened the panel to invite participants from backgrounds other than child oral health but with experience of family health behaviours and interventions such as those pertaining to nutrition [25, 26].

Potential participants in each expert panel were sent an invitation email with an information sheet and a link to the online survey in Microsoft Teams. A reminder email was sent out 2 weeks following the initial invitation to those who had not yet completed each initial survey as is recommended [24, 27]. The initial surveys were open for 5 weeks before responses were aggregated. Written informed consent was obtained from all participants prior to taking part in the research. Snowballing [28] was employed whereby those invited to take part were asked for suggestions of colleagues with expertise who may be willing to be approached.

#### 2.5.1 Delphi Rounds 1 and 2: Initial Survey and Iteration on Home Toothbrushing Barriers

Round 1 of the modified Delphi survey presented 13 barriers to participants for initial consideration. These were categorised into three areas: child, parent/carer and family environment‐related factors. Data S3 contains a description of each barrier as presented to participants. Participants were asked to rate each barrier based on their level of agreement that the barrier should be included in the intervention using a five‐point Likert scale (1—Strongly Disagree, 2—Disagree, 3—Neither Agree nor Disagree, 4—Agree, 5—Strongly Agree). They were also asked to list any potential missing barriers.

In Round 2 participants were re‐presented with barriers, this time indicated for inclusion/ exclusion based on the round 1 aggregated scoring. Inclusion was set via convention for Delphi studies [13, 18] at 75% of participants initially selecting agree/ strongly agree that the barrier was a priority for inclusion in the home‐based toothbrushing intervention, giving regard to the target population of families (deemed to be in need of additional support).

For the inclusion set, participants were asked for a yes/no response as to whether they felt the barrier list was comprehensive and whether barriers did not overlap too much.

#### 2.5.2 Delphi Rounds 3 and 4: Initial Survey and Iteration on Behaviour Change Techniques and Implementation of the Tool

Rounds 3 and 4 were surveys on appropriate behavioural techniques and delivery of the intervention. Using the same Likert agreement scales, opinions were sought on various aspects of the overall approach, on specific behaviour change theories employed and on design and implementation (e.g. hand‐held devices or tablets versus physical cards). In Round 4 participants were presented with a summary of the level of agreement from the previous round and were asked via yes/no questions whether they agreed that the overall approach and behaviour techniques were appropriate to include in the intervention.

Anonymous rating data from all surveys were exported from the online survey software into a Microsoft Excel spreadsheet scored securely on the University server.

### 2.6 Interview Procedures

Participants for the qualitative interviews were recruited via an initial approach to the Childsmile Programme management team who provided contact details based on Data Protection arrangements in the ethical approval for those that might be interested in taking part. DHSWs were then contacted by the research team via email and provided with a participant information sheet.

Interviews were arranged with those consenting and carried out in person (*n* = 5) or over Microsoft Teams (*n* = 7) according to local Covid‐19 travel restrictions in place at the time, with participants being able to choose their preference. The average duration of interview was 80 min, with interview durations ranging from 58 to 134 min. Interviews were carried out by EF (dentist and PhD candidate). The interview guide can be found in Data S4.

### 2.7 Analysis

The results from the Delphi surveys are presented via descriptive statistics of the percentage agreement and median scale scores for items with minimum and maximum scores and Interquartile Range. All Delphi rounds had ‘free response’ items for participants to explain their agreement ratings or suggest alternative barriers, approaches etc. These are used for illustration where appropriate.

Interview analysis was carried out by EF (dentist and PhD candidate and AR, Psychologist) using Template Analysis techniques. This is a pragmatic approach to qualitative studies employed where there are pre‐existing questions that are aimed at eliciting specific responses [29]. These groups of responses on particular issues give rise to a priori themes (areas identified in advance as pertinent to the research question). TA is therefore more deductive than thematic approaches that are more generative, drawing from Grounded Theory [30] and privileging data immersion and theory generation. TA analysis allows for a coding hierarchy with a reasonably high level of structure alongside the flexibility to adapt where required for a particular study [31]. Coding takes place within the initial themes (the template) and can be used to revise them if necessary.

Four a priori top‐level themes were generated from structured interview questions and their responses. All interviews were audio recorded and transcribed for analysis. Initial coding (labelling of textual units) was conducted by EF and short definitions of each subtheme produced to populate the template. New themes may emerge where codes do not fit existing ones. This proved necessary with a new emergent theme around children with additional support needs (see results).

Reflexivity was provided by EF and AR coding different interviews separately and comparing similarities and differences. Initial templates included queries on ambiguous or difficult‐to‐assign codes and were shared for discussion and resolution with the wider team.

**FIGURE 1:**
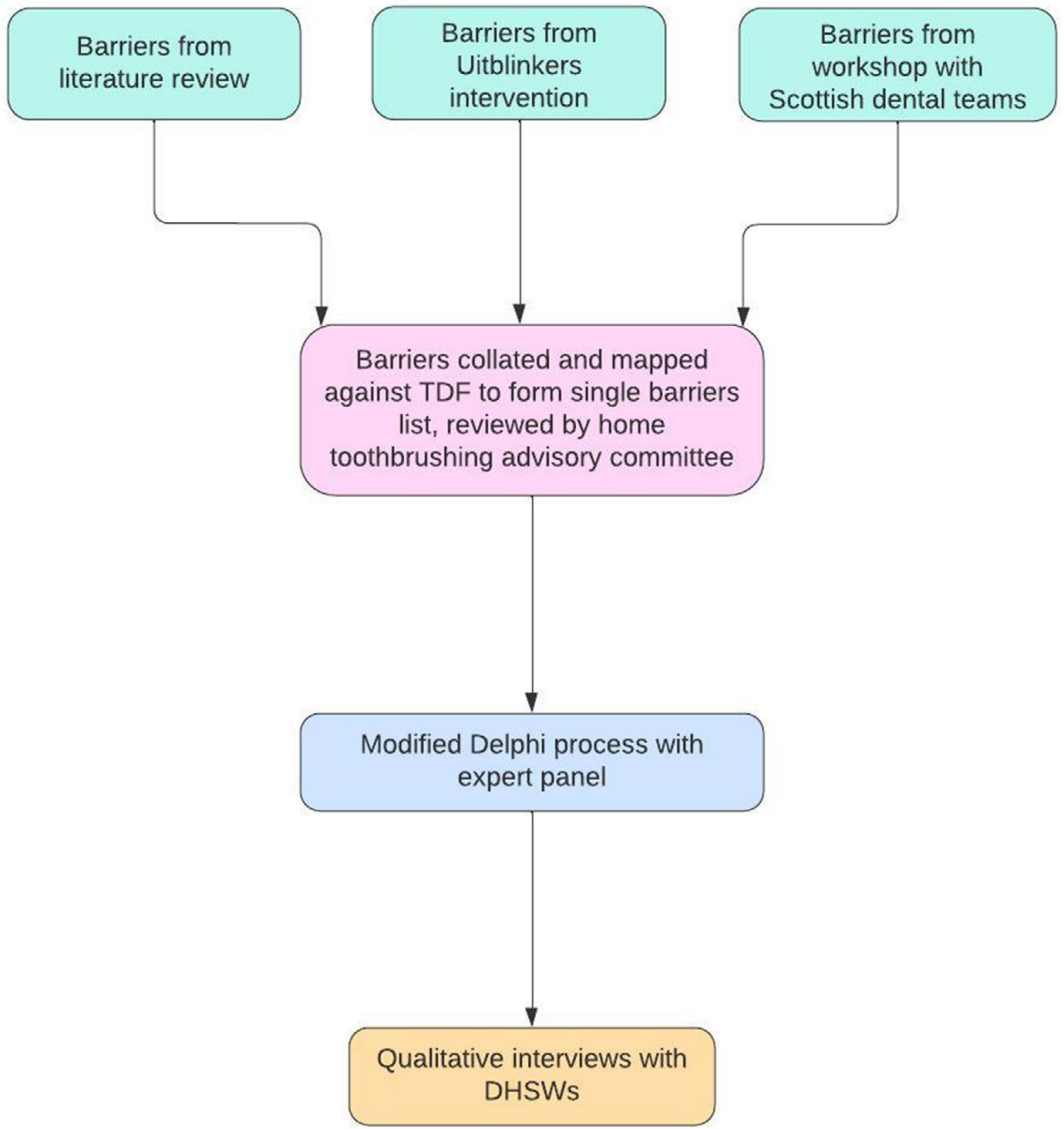
Flow diagram outlining the method process.

The method process is shown in the flow diagram in Figure 1.

## 3 Results

### 3.1 Delphi Study Results

A total of 21 participant researchers and practitioners from dentistry and the dental team, public health and behaviour change completed the modified Delphi Round 1 on toothbrushing barriers and 18 (86%) completed the follow up Round 2. There were also 21 responses to Round 3 on behaviour change and implementation and 17 (81%) responded to the iterative consensus exercise in Round 4.

### 3.2 Research Aim 1: Identifying the Barriers That Parents/Carers in Need of Extra Support Face in Implementing Supervised Toothbrushing

Table 2 shows initial agreement on including proposed barriers (in order of the proportion of positive responses with smaller range indicating consensus).

Three initial barriers did not meet the 75% level of consensus agreement in Round 1 and were proposed for exclusion in Round 2: *External input; Child too tired/child falling asleep; Parent/carer knowledge*. As might be expected given these had still had majority support in Round 1 (72%–62%) there was some disagreement in Round 2. Scores ranged from strongly disagree to strongly agree, and the only slight majority for exclusion was *External input* at 56%. Following discussion with the home toothbrushing advisory group, all initial barriers were incorporated via rationalising the list (Table 2) and a set of 11 barriers is thus proposed for inclusion in the intervention (see discussion).

At Round 2, 17/18 (94%) of participants agreed that the list formed a comprehensive set of child home toothbrushing barriers faced by families who may be in need of additional support, as one anonymous respondent explained: “*all barriers appear to be covered and these barriers I have faced on a few occasions*” [Delphi ID 2].

In terms of barriers being exclusive 16/18 (89%) felt there was overlap. This wasn't in itself felt to be an issue: *They overlap because they have common causes. Parents who are less confident in their abilities to look after children's teeth may be more likely to be less confident in their abilities to manage difficult child behaviour and may also be more likely to have unstable routines*. [Delphi ID 1].

### 3.3 Research Aim 2: Identifying Appropriate Behaviour Change Techniques to Address Barriers

**FIGURE 2:**
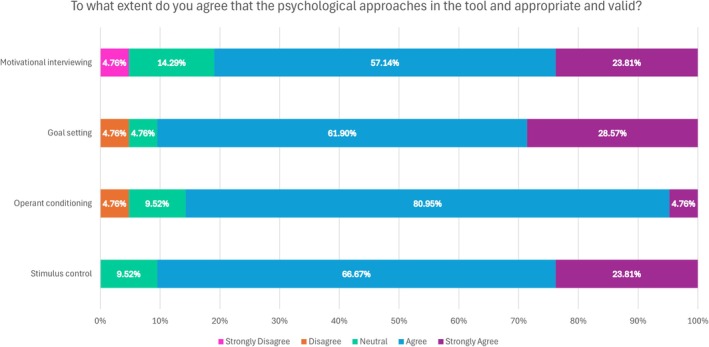
Panel responses (*n* = 21) on the appropriateness and validity of the psychological approaches in the proposed tool.

In Round 3 the use of cards for delivering advice was supported with 15/21 agreeing and 3/21 strongly agreeing (86% in total) that families would be receptive. Participants were asked to what extent they agreed that the psychological approaches in the tool were appropriate and valid. Results are shown in Figure 2. Figure 2 shows strong support with a maximum of one participant disagreeing/ strongly disagreeing across the techniques. As one expert respondent said: *I think using the MI approach with stimulus control as the change theory should be satisfactory. I think using the principles of operant conditioning with very specific examples (not mentioning the aspect of punishment) might elicit change as well*. [Delphi ID 11].

The use of cards was supported with 15/21 respondents agreed that families would be receptive to advice if delivered in this way (71%) with three strongly agreeing. Only one person disagreed and two were neutral. Round 4 was condensed into a simple yes/no response as to the appropriateness, and agreement was: Stimulus Control and Goal Setting (16/17; 94%); Operant Conditioning (15/17; 88%) and Motivational Interviewing (14/17; 82%).

### 3.4 Research Aim 3: Making Recommendations for Design of a Co‐Produced Home‐Support Tool and Identifying Facilitators for Implementation in Practice

Table 3 presents the level of agreement in Round 3 for various aspects regarding delivery of the intervention in the home setting. It can be seen that implementation is deemed realistic but that training is indicated. Physical cards are preferable, supplemented with reminder materials and the main disagreement is on whether remote/ digital delivery could be effective.

There was strong consensus and no change to these responses in Round 4; only one respondent disagreed that the physical cards were the preferred delivery method in the home (1/17; 6%; see interview responses for more detail): *I believe this is a good way of presenting a set of barriers. Parents again will feel seen and heard and are reassured that they are not the only one with these problems*. [Delphi ID 3].

### 3.5 Interview Results With DHSW (Aim 1, 2 and 3)

Table 1 outlines the characteristics of the 12 DHSW interview participants. Below are 5 main themes that emerged from the interviews with DHSWs on the proposed toothbrushing barriers, behaviour change techniques and considerations for implementation, using template analysis. Data S5 shows subthemes under top‐level themes 1–5 with descriptions and illustrations from participant interviews.

**TABLE 1:** Characteristics of Dental Health Support Worker (DHSW) participants in qualitative interviews.

Participant	Gender	Length of time as a DHSW	Interview format
Greater Glasgow and Clyde (Abbreviation Glasgow)			
1	F	15 years	In person
2	F	15 years	In person
3	F	14 years	In person
4	F	8 years	In person
5	M	4 years	In person
Highland			
6	F	13 years	Online (MS Teams)
7	F	10 years	Online (MS Teams)
8	F	7 years	Online (MS Teams)
Ayrshire and Arran (Abbreviation Ayrshire)			
9	F	10 years	Online (MS Teams)
10	F	8 years	Online (MS Teams)
11	F	6 years	Online (MS Teams)
Tayside			
12	F	10 years	Online (MS Teams)

**TABLE 2:** Initial and final barriers from Delphi Rounds 1 and 2 on priority for inclusion in the home‐based toothbrushing intervention.

Number (*E* = initially excluded)	Barrier	Percentage agreement (% respondents agreeing or strongly agreeing)	Median	Min	Max	Q1^c^	Q3
1	Difficult child behaviour/non‐compliance	100% (*n* = 21)	5	4	5	5	5
2	Structures and routines	100% (*n* = 21)	5	4	5	5	5
3	Parent/carer capability	95% (*n* = 20)	5	3	5	5	5
4	Social setting and influences	95% (*n* = 20)	5	3	5	4	5
5	Parent/carer attitudes and motivation	86% (*n* = 18)	5	3	5	4	5
6	Time constraints	86% (*n* = 18)	4	2	5	4	5
7	Cultural barriers	86% (*n* = 18)	4	2	5	4	5
8	Child appears upset	81% (*n* = 17)	4	2	5	4	5
9	Parent/carer self‐care	81% (*n* = 17)	4	2	5	4	5
10	Family resources	76% (*n* = 16)	4	3	5	4	5

75% agreement cut‐off							
E1	External input^b^	72% (*n* = 15)	4	2	5	3	5
E2	Child too tired/falling asleep^a^	67% (*n* = 14)	4	2	5	3	4
E3	Parent/carer knowledge^b^	62% (*n* = 13)	4	2	5	3	5

^a^ Combined with barrier 8 in final set.

^b^ Combined together to form new included barrier 11 in final set.

c The lower quartile, or first quartile (Q1), is the value under which 25% of data points are found when they are arranged in increasing order. The upper quartile, or third quartile (Q3), is the value under which 75% of data points are found when arranged in increasing order.

**TABLE 3:** Results from Delphi Round 3 on delivery of the proposed intervention in the home.

Aspect of delivery	Percentage agreement (% respondents agreeing or strongly agreeing)	Median	Min	Max	Q1^a^	Q3
‘Support workers would need brief training in psychological theory to deliver the intervention’	95% (*n* = 20)	4	3	5	4	5
‘Online delivery such as via hand‐held devices or tablets would be better than physical cards’	24% (*n* = 5)	3	2	5	3	3
‘This could realistically be delivered in the home setting’	95% (*n* = 20)	4	3	5	4	5
‘Resources/materials to leave with families (e.g. reminders, diaries) would help’	100% (*n* = 21)	4	4	5	4	5
‘This could be delivered effectively remotely (e.g. video calls)’	62% (*n* = 13)	4	2	5	3	4

a The lower quartile, or first quartile (Q1), is the value under which 25% of data points are found when they are arranged in increasing order. The upper quartile, or third quartile (Q3), is the value under which 75% of data points are found when arranged in increasing order.

### 3.6 Theme 1 the Barriers Present an Exhaustive Set and Are Valid From Staff Experience With Families (Aim 1)

The barriers as presented were deemed valid in that they match staff experience of the issues they see families face when they carry out home visits. They also present complete coverage of barriers from staff experience (although see emergent Theme 5).

### 3.7 Theme 2 Motivational Interviewing Is Appropriate and Fits With Usual Practice (Aim 2)

The general approach of a Motivational Interview was deemed appropriate in two ways. First it builds rapport through opening in a positive manner and importantly it is also aligned with current support worker practice and training. As one respondent reports: *You can't just go in and point the finger at people, do you know, that doesn't work here* [Participant 5, Glasgow, 4 years experience].

### 3.8 Theme 3 the Included Behaviour Change Techniques Are Workable (Aim 2)

There was general positive reaction to the behaviour change techniques and theories employed. DHSWs liked the use of card selection as a prompt to the conversation and the employing of the indicated tips and strategies. The visual nature of the cards was felt to be inclusive and engaging. One subtheme emerged with strong support which was to find a way to leave supporting material after discussions as a reinforcement technique.

### 3.9 Theme 4 the Tool Is Generally Feasible Within the Operation of Childsmile Home Visits (Aim 3)

There was a general feeling the tool could work in home visits with some provisos. In this regard a physical tool was preferred with electronic devices being less reliable and a smaller size on balance was deemed important due to portability and the ability to incorporate in an existing A5 pack used by DHSWs.

### 3.10 Theme 5 the Tool Is Not Less Applicable for Children With Additional Support Needs (Aim 3)

The tool was not specifically designed for parents of children with additional support needs. However, interviews illustrated that this vulnerable group, particularly with respect to Autism Spectrum Disorder, are increasingly encountered in referred families and seem to have discrete barriers such as resistance to certain textures. The tool might need future amendment (or a similar tool might be developed) to provide tailored support for this group (see discussion).

### 3.11 Qualifiers and Exceptions to the Template of Themes and Subthemes

Finally, as would be expected the participants do not speak with an entirely uniform voice across these themes and there are some qualifiers and exceptions to general positive reactions to the proposed tool.

As is common in health services for vulnerable populations it was felt the tool might work better for those in slightly less need of support: *I think it sounds good. I think it's hard because I don't know, will the people that change be the people that you're wanting to get to change?* [Participant 12, Tayside, 10 years experience]. There were some queries about missing deeper social barriers related to parents' drug and alcohol misuse (see discussion).

DHSWs were clear that utilising the tool would have to be assessed on a visit‐by‐visit basis when it was felt families might be receptive. Future testing in the ‘real world’ (see discussion) will further illustrate implementation issues, for example: *Not everyone wants you in their house either […] I've done a home visit at the front door. It's taken three minutes […] my shortest visit*. [Participant 6, Highland, 13 years experience]. The home visits also vary across the country and some felt that even when families are accepting they might not have the time to deliver the intervention alongside routine advice: *I don't know that I would go into such depths when I'm going in to do a visit. Usually what I do is, I've got a sort of set piece you know?* [Participant 2, Glasgow, 15 years experience].

Follow‐up is an important aspect of the overall tool approach but there are issues with capacity that has seen this sometimes erode: *have always done a follow up call with parents […] But the way it changed over the years was we didn't have the capacity to do that and we didn't have the time to do that […] it would need to be checked on capacity. Would we have the capacity to?* [Participant 9, Ayrshire, 10 years].

The tool design was presented whereby the barriers are set out for parents to choose/ self‐identify with. In principle people felt this was a good idea (see Data S5) but there are practical and potentially social issues that might mean some flexibility is required in use: *[…] when I sit in someone's house I have my bag, I put down. I have my paperwork in front of me […] so I would have my bag on my knee. I would never use somebody's table. […] To be honest with you [name] I think I would only be taking the one card out, unless I really thought I needed two or three. I just wouldn't take them all out and put them on a table and go, let's play a game here, because it's kind of patronising, I feel*. [Participant 6, Highland, 13 years experience].

## 4 Discussion

This study aimed to modify an existing home toothbrushing support tool for use within.

Scotland's National Oral Health Programme for Children, Childsmile, by ensuring it targeted the barriers that parents/carers in need of extra support face in implementing supervised toothbrushing. Further aims were to identifying appropriate behaviour change techniques to address these and identify facilitators for future implementation and testing in practice.

Key findings include a set of 11 barriers and associated behavioural tips/strategies validated by an expert panel and users of the proposed tool, and a preference for a physical set of pictorial cards. The tool was deemed suitable for the home setting with some provisos, mainly in terms of the context of use (such as available time and resources). Finally, there may be a requirement for a tool tailored specifically to children with additional support needs.

Inequality remains a major challenge worldwide and in the UK for child oral health and additional preventive care for children at higher risk is recommended in recent guidance which recognises people face multiple barriers and that motivational support towards, building trust and rapport, is necessary [32, 33]. The agreed‐upon tool is built around a Motivational Interview for which there is some evidence of efficacy above traditional oral health education in promoting good oral health behaviour and sustaining changes [34, 35].

The final proposed barriers to be addressed through the tool draw from an existing dental practice‐based tool employed in the Netherlands [8] and were also mapped against a well‐known determinant framework of influences on health behaviours. This supplements the overall motivational intent with addressing capability (including helping parents address child compliance) and opportunity (addressing barriers to do with time, routines and social norms and culture) [36]. All of the final included barriers were reported in the results of a recent systematic review conducted to investigate the barriers (and facilitators) to toothbrushing behaviours in the home by parents of young children [19] and the highest priority barriers in terms of behavioural regulation and structures and routines match the most common in this review which would indicate content validity.

Two broad psychological theories guide the tips and strategies to address the barriers: Stimulus Control targeting opportunity barriers in the physical and social environment and Operant Conditioning seeking to positively reinforce (and avoid negative reinforcement) in overcoming capability issues and fostering parent/carer confidence [37]. The evidence for affecting health behaviours through Operant Conditioning is mixed in interventions for young people and adolescents [38] but there is little research on supporting parents/carers to employ these techniques in helping their children's uptake of good habits and future evaluation will certainly be necessary.

The final set of barriers included an agreed barrier in terms of information, namely the interaction between external input/ patient knowledge. Knowledge alone is known to have limited effect in bringing about behaviour change [39, 40, 41] but conversations in this regard may be a foundation for discussions on the important aspects of how to support the behaviour in context [42].

Finally, there are previously reported additional behavioural and sensory barriers faced by children with additional support needs, principally around Autistic Spectrum Disorder [43]. Home support for families and children in this regard emerged as a theme, and further work will be required to examine how suitable the proposed tool is beyond the initially targeted population.

## 5 Strengths and Limitations

Strengths of the study included the involvement of support workers (users) in designing the research [44] and the recruiting of a varied and experienced expert panel. Staff participants represent the users of the proposed tool, which has the support of the necessary National Health Service (NHS) functions to enable further testing, implementation and evaluation. Involving end‐users in design of tools in healthcare is an embedded principle for medical devices and there are calls for structured approaches to widen this to all practice‐based tools [45].

The study is an international collaboration drawing from previous validation of the overall approach, content and format of a validated tool in the Netherlands but it has also employed mixed‐methods in the first attempt to develop consensus on a targeted set of toothbrushing barriers and behavoural techniques for use in a home‐based toothbrushing intervention for families referred for support.

There are some limitations of the study. Strengths of the Modified Delphi technique include anonymity of response reducing bias [46] and the validation of responses through feedback/iteration rounds. However, despite its wide application in health care, there are some concerns. There is little to no guidance on how to develop questions/survey items, how to select panel members (and how many) on what criteria, how many rounds or iterations are appropriate, or on consensus thresholds [47]. There may be bias in the final results introduced by any or all of these matters.

However, the propositions put forward came from a careful interrogation of previous evidence and from the validated *Uitblinkers* tool and a toothbrushing advisory group assessed the initial barrier list for completeness. The panel were given a clear opportunity to put forward opinions, qualifications and alternatives and Delphi consensus was also tested in in‐depth qualitative interviews allowing for data triangulation. Another limitation of this study is that parents and carers were not included in the Delphi panel to identify toothbrushing barriers. Therefore, some relevant barriers might have been missed or falsely included as relevant. However, the lists of barriers included in the Delphi study were based on the *Uitblinkers* intervention, which identified toothbrushing barriers using focus groups with parents [37, 48] and on literature of studies conducted with parents and carers [42, 49, 50, 51, 52, 53, 54, 55, 56, 57, 58, 59, 60, 61, 62, 63, 64]. For the Delphi panel, researchers and clinicians with research or practical expertise with child toothbrushing in the home setting were specifically selected, since they could also provide advice on barriers, behaviour change techniques and implementation tools they know to be useful.

The qualitative interview phase employed a relatively deductive method to further explore the tool development with staff. This can mean some emergent themes from experience might be suppressed but is pragmatic when there are clear answers required to specific questions. Furthermore, the underpinnings of inductive thematic analysis that purports to have no preconceived codes or constructs has been extensively critiqued; it was deemed better to make these explicit in advance [65].

## 6 Conclusions and Next Steps

This study adds to knowledge by describing expert consensus on barriers to children's home toothbrushing faced by families referred for extra support and ways to address these in a dedicated support tool based on a Motivational Interview. It supports national policy and practice in Scotland by drawing from international evidence and seeking the views of support workers who will deliver any final intervention.

As part of the embedded Childsmile research and evaluation programme, a prototype tool is now being tested in simulated settings with parents and in the home setting across Scotland in a stepped‐wedge cluster randomised trial, clustered by geographical area and following training for support workers. This is indicated when the intent is that all clusters ultimately receive the intervention [66]. The primary outcome will be toothbrushing frequency obtained from Child Health Surveillance data.

## Conflicts of Interest

The authors declare no conflicts of interest.

## Supporting information


Data S1.

Data S2.

Data S3.

Data S4.

Data S5.

## Data Availability

The data that support the findings of this study are available on request from the corresponding author. The data are not publicly available due to privacy or ethical restrictions.
