# Supplementary material for: Developing a Prototype Home‐Based Toothbrushing Support Tool for Families in Scotland: A Mixed‐Methods Study With Modified Delphi Survey and Semi‐Structured Interviews
Source: Community Dent Oral Epidemiol. 2025 Feb 12;53(3):296–306. doi: 10.1111/cdoe.13031 (PMC12064878; doi:10.1111/cdoe.13031)

Supplementary File 1


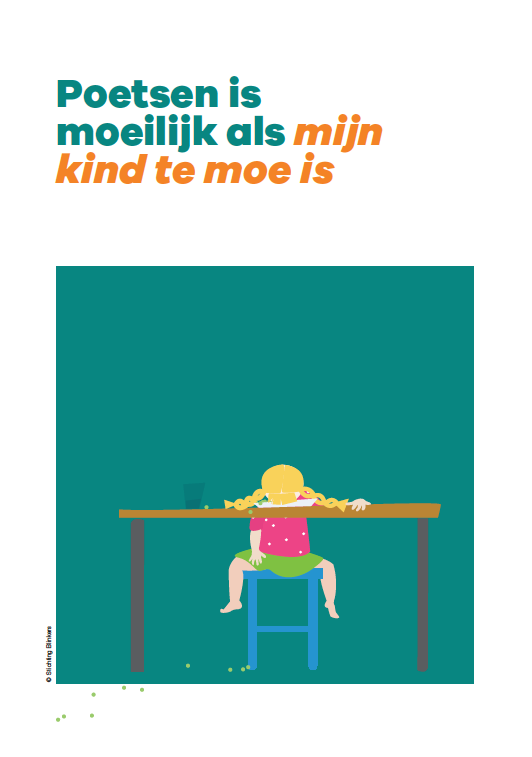
Uitblinkers barrier card: Toothbrushing is challenging when my child is too tired

Uitblinkers barrier card: Toothbrushing is challenging when I am stressed or pre-occupied


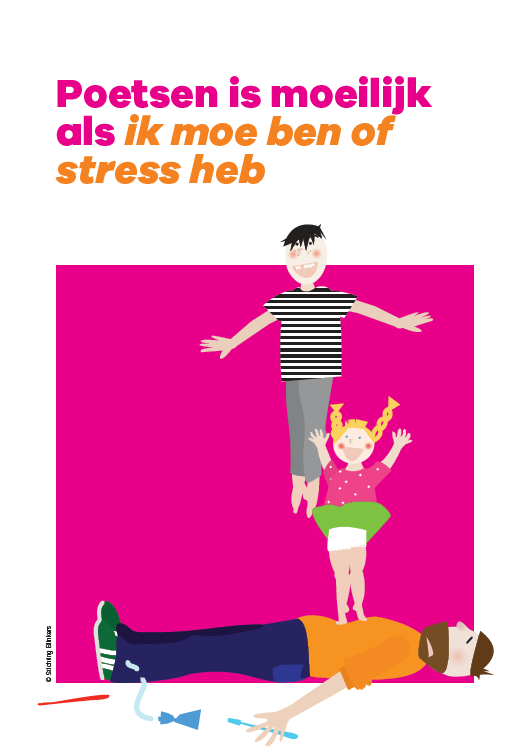

Supplement: Supplementary file 1 — Data S1. [file CDOE-53-296-s003.docx]
