# Supplementary material for: Developing a Prototype Home‐Based Toothbrushing Support Tool for Families in Scotland: A Mixed‐Methods Study With Modified Delphi Survey and Semi‐Structured Interviews
Source: Community Dent Oral Epidemiol. 2025 Feb 12;53(3):296–306. doi: 10.1111/cdoe.13031 (PMC12064878; doi:10.1111/cdoe.13031)
Supplement: Supplementary file 2 — Data S2. [file CDOE-53-296-s001.docx]

Supplementary file 2

**Barriers from literature review**

| **Paper** | **Description** | **Barriers to toothbrushing** | **Theoretical Domains Framework domains** |
| --- | --- | --- | --- |
| Duijster et al (2015)  (50) | - Dutch study with Dutch parents of low and high socioeconomic status and parents from Turkish and Moroccan origin - 39 parents of 7 year old children - Focus groups | - External locus of control – parents did not believe oral hygiene efforts could prevent their child from getting tooth decay. | - Beliefs about capabilities |
|  |  | - Difficult child behaviour and non-compliance e.g. resistant behaviour, tantrums, pain during teething, tiredness of child | - Emotion - Behavioural regulation |
|  |  | - Time constraints/busy schedules – especially in the mornings | - Environmental context and resources |
|  |  | - Parental knowledge – some parents insecure about details of knowledge | - Knowledge |
|  |  | - Complicated advice – some parents found difficult to adhere | - Environmental context and resources |
| Elison, et al (2014)  (51) | - 16 first time mothers of children aged 24-30 months living in one of two areas of Greater Manchester with the worst rates of dmft in under 5s. - Recruited from local childcare services - Qualitative interviews at participants’ home | - Perceived maternal self-efficacy for tooth-brushing – important to feel confident to establish a toothbrushing routine in order to do so | - Beliefs about capabilities |
|  |  | - Stress related to difficulties experienced when trying to establish toothbrushing routine | - Emotion |
|  |  | - Ability to remember to brush child’s teeth – especially at night | - Memory, attention and decision processes |
|  |  | Child behaviours:   - Child wants to brush themselves, grabbing toothbrush - Child dislikes toothbrushing/taste resulting in non-compliance - Refusal to open mouth - Child sleeping - parent doesn’t want to wake to brush | - Skills - Behavioural regulation |
|  |  | - Lack of support from others - Lack of practical advice from healthcare professionals | - Social influences |
|  |  | - Family history – parents own experience as a child – what is ‘normal’ to them | - Nature of the behaviours |
| Marshman et al (2016)  (42) | - 27 interviews with parents living in Bradford and Barnsley, mostly living in deprived areas - purposively sampled through a maximum variation approach to ensure inclusion of the following: parents (or caregivers with parental responsibility) living in deprived areas; of different sexes, ethnicities, and first languages; with children of varied ages and dental attendance patterns, with and without dental caries | - Knowledge – not aware of guidance to help brush until child is 7 years old | - Knowledge |
|  |  | - Social influences e.g. grandparents letting children get away with not brushing | - Social influences |
|  |  | - Mothers perceived (by both mothers and fathers) to have overall responsibility for child toothbrushing | - Social/professional role and identity |
|  |  | - Manual skills – worried about hurting their child when brushing – especially when first starting as babies - Child behaviour regulation – lack of parenting skills to manage behaviour of unwilling child – often encountered challenging behaviour when child reaches 18-24 months – crying, keeping mouth closed, wanting to brush by themselves | - Skills |
|  |  | - Beliefs about capabilities in managing their children’s behaviours – lack of control | - Beliefs about capabilities |
|  |  | - Breaks in routine e.g. weekends, school holidays, sleepovers - Stressful environment/circumstances that make day to day life a struggle e.g. several young children to look after, unemployment, debt, drug use, single parenthood, and domestic abuse | - Environmental context and resources |
|  |  | - Prioritising activities/toothbrushing taking place at busy times e.g. getting children to bed, eating breakfast, leaving for school | - Behavioural regulation |
| Huebner et al (2010)  (52) | - 44 parents of infant/preschool children interviewed in rural southwest Washington State - Participants were clients of 1 of 3 early childhood education programs in the community that served low-income families with infants or preschoolers. | - Oral health beliefs – some false beliefs, worry about damaging teeth if brush too much | - Knowledge |
|  |  | - Social norms – lack of social norm or other support for twice daily brushing - Emotional reactions – not wanting to upset child or, in turn, themselves | - Social influences - Emotion |
|  |  | - Self-standards – feeling that brushing once a day is sufficient | - Beliefs about consequences |
|  |  | - Self-efficacy – parents don’t know how to overcome self-reported barriers | - Beliefs about capabilities |
|  |  | - Skills – not making twice daily brushing a habit | - Skills |
|  |  | - External constraints – struggles with fussy/moody child, lack of time in rushed schedule, particularly in the morning | - Environmental context and resources |
| Finlayson et al (2019)  (53) | - 24 interviews with parents of children aged between 6 months – 4 years - Parents enrolled in the home visit component of the Early Head Start programme in LA | - Child resistance, stage of development – increasing sense of independence - Deviation from regular routine – child sickness, tiredness, not wanting to wake child to brush teeth | - Environmental context and resources |
|  |  | - Different caregivers carrying out toothbrushing e.g. some fathers more relaxed about toothbrushing, less strict with the routine | - Social influences |
|  |  | - Knowledge – many parents unsure when children should brush independently – answers ranged from 1 to 5 years of age | - Knowledge |
|  |  | - Lack of effective solutions to overcome difficulties led to abandoning brushing and waiting until child is cooperative | - Skills |
| Trubey et al (2014)  (54) | - 15 parents of children aged 3-6 years, semi-structured interviews - Purposive sampling on the basis of child’s involvement in a national, school‐based toothbrushing scheme called Designed to Smile - Programme is run in nurseries and schools in areas of high soci-economic deprivation in Wales | - Disrupted routines – mornings often ‘hectic’ but more stable routine. Evening routine subject to change e.g. work schedules, after school clubs – toothbrushing could be a struggle and so, often missed | - Environmental context and resources |
|  |  | - Knowledge/attitude - Some parents did not see importance of brushing in the evening if brushing in the morning anyway | - Knowledge |
|  |  | - Toothbrushing norms – parents compared their habits to others. - Parents who believed that very few other parents brushed twice per day, thought that the message about what you should do (twice daily brushing) was not credible. - Some parents who brushed less frequently sceptical that others brushed more regularly. | - Social influences |
|  |  | - Some parents content with less frequent brushing because they believed other parents were acting similarly | - Motivation and goals |
| Aljafari et al (2014)  (55) | - 29 interviews with parents of children aged 3-10 years who have had teeth extracted under general anaesthetic | - Difficulties maintaining toothbrushing routine due to factors such as: - Time constraints - Number of children - Children’s attitudes and brushing skills | - Environmental context and resources - Skills |
|  |  | - Lack of practical toothbrushing advice | - Social influences |
| Amin, et al (2009)  (56) | - 26 interviews with 18 parents of children who had been referred to a specialty paediatric dental practice due to young age of child and extent of treatment needed - Children aged between 2.5 and 6 years old | - Resistance from child | - Skills |
|  |  | - Stresses of daily life | - Environmental context and resources - Emotion |
| Finlayson et (2019)  (57) | - 6 focus groups and 1 interview with lower income Hispanic mothers of preschoolers aged 3-5 years (n=36 total participants) - Community health clinic on California-Mexico border | - Lack of time/oral health not a priority | - Environmental context and resources - Behavioural regulation |
|  |  | - Uncooperative child | - Skills |
| Lotto et al (2020)  (58) | - Focus groups with parents of children who attended paediatric dental clinics - 17 participants with mean age of 34.9 years and income of US$ 591.17 | - Busy lives/lack of time | - Environmental context and resources |
| Naidu et al (2012)  (59) | - 3 focus groups with 18 parents of children attending preschool | - Difficulties achieving night time brushing due to child falling asleep soon after last meal | - Nature of the behaviours |
|  |  | - Lack of support | - Social influences |
| Reidy et al (2001)  (60) | - 7 community focus groups with 5 ethnic populations on the island of Saipan, self-governing US island in western Pacific Ocean - 41 mothers and grandmothers of who had between 1 and 7 children/grandchildren, age range from 3 months to 20 years. Majority had 3 children under 4 years old. - Mix of working and non-working mothers and job types | - Difficulty brushing a baby or young child’s teeth – resistance from child | - Skills |
| Virgo-Milton et al (2016)  (61) | - Semi-structured interviews with 32 mothers of young children age 6 months or older - Variety of demographic variables (socioeconomic, family size) | - Lack of time in busy schedules | - Environmental context and resources |
|  |  | - Child uncooperative | - Skills - Behavioural regulation |
| Mofidi et al (2009)  (62) | - Focus groups with parents (n=22) and pregnant women (n=13) enrolled in the Early Head Start programme - Purposive sample to represent four major ethnic groups | - Lack of awareness of importance of deciduous teeth and caring for oral health of young children | - Knowledge - Beliefs about consequences |
|  |  | - Busy routines and other priorities | - Environmental context and resources |
|  |  | - Child uncooperative | - Skills, - Behavioural regulation |
|  |  | - Knowledge – unsure how to brush a young child’s teeth | - Knowledge |
| Prowse et al (2014)  (63) | - Focus groups with parents/carers (n=40) of children <6 years old - Four different cultural groups living in Manitoba, Canada | - Child uncooperative | - Skills - Behavioural regulation |
|  |  | - Lack of time due to parent tiredness | - Environmental context and resources - Memory, attention and decision processes |
| Van Nes et al (2018)  (64) | - Focus groups (n=16) and interviews (n=13) with mothers of Dutch-Moroccan preschool children in the Netherlands | - Difficulties supervising toothbrushing due to child resistance | - Skills - Behavioural regulation |
|  |  | - Daily morning chaos and fatigue in the evening | - Environmental context and resources |
| Weistein et al (1999)  (65) | - Interviews with Native American mothers (n=62) of children age 0-3 years in the USA | - Expectation that children should brush their own teeth from one year of age | - Knowledge - Beliefs about capabilities |
|  |  | - Mulptiple children to care for | - Environmental context and resources |
|  |  | - Difficulties with toothbrushing when child reached 2 years old | - Behavioural regulation |
| Zeedyk et al (2005)  (66) | - Observation of videotaped toothbrushing sessions with 18 families in the UK - Average age of child=2.5 years old | - Child cooperation/lack of concentration | - Skills - Behavioural regulation |
|  |  | - Parents’ lack of confidence | - Beliefs about capabilities |
