# Supplementary material for: Developing a Prototype Home‐Based Toothbrushing Support Tool for Families in Scotland: A Mixed‐Methods Study With Modified Delphi Survey and Semi‐Structured Interviews
Source: Community Dent Oral Epidemiol. 2025 Feb 12;53(3):296–306. doi: 10.1111/cdoe.13031 (PMC12064878; doi:10.1111/cdoe.13031)
Supplement: Supplementary file 3 — Data S3. [file CDOE-53-296-s005.docx]

Supplementary File 3

Description of barriers used in Delphi Round 1:

**Child related barriers**

- Difficult child behaviour/non-compliance (e.g. lack of child co-operation, child refusal, child restlessness)
- Child too tired/child falling asleep
- Child appears upset - (e.g. child in tears/crying, shows discomfort)

**Parent/carer related barriers**

- Parent/carer knowledge (e.g. knowledge of decay, of fluoride, of need for twice daily toothbrushing, of age to start toothbrushing)
- Parent/carer capability (e.g. manual brushing skills, skills in managing child behaviour or resistance, not confident in their own ability to supervise effective toothbrushing, asserting parental brushing when child wants to brush themselves)
- Parent/carer attitudes or motivation (e.g. - parent doesn’t see importance, parent doesn’t want to force child, parent has given up or feels helpless to avoid decay)
- Parent/carer self-care (e.g. parent too tired, parent stressed, parent unfit or unwell)

**Family environment related barriers**

- Time constraints (e.g. other priorities, busy schedules)
- Social setting and influences (e.g. influence of others; lack of support from family and friends, unhelpful norms, poor role models, parent’s own experience as a child)
- Structures and routines (e.g. disrupted routine, lack of routine; multiple caregivers, child lives between houses, multiple children to care for, parents often interrupted and/or forget)
- Family resources (e.g. financial problems, lack of toothbrushes/toothpaste, no appropriate space for brushing)
- External input (e.g. complicated or confusing advice from professionals, lack of instruction, access to services)
- Cultural barriers (e.g. norms regarding toothbrushing practices, language barriers, access to culturally appropriate materials and resources)
