# Supplementary material for: Developing a Prototype Home‐Based Toothbrushing Support Tool for Families in Scotland: A Mixed‐Methods Study With Modified Delphi Survey and Semi‐Structured Interviews
Source: Community Dent Oral Epidemiol. 2025 Feb 12;53(3):296–306. doi: 10.1111/cdoe.13031 (PMC12064878; doi:10.1111/cdoe.13031)
Supplement: Supplementary file 4 — Data S4. [file CDOE-53-296-s004.docx]

Supplementary file 4

**Dental Health Support Worker Interview Guide**

- Tell us a bit about yourself and your work in oral health support?
  - E.g. experience, role, location
- What do you think are the main issues facing parents of young children with regards to caring for their child’s oral health?
  - Difficulties with toothbrushing?
    - Prompts from modified Delphi exercise
- What if anything have you tried with parents/ carers before?
  - What worked, what didn’t, why/ why not?

[From here, introduce concept of intervention – bit of background about Uitblinkers, what it is, what we are doing. Opening conversation with positive behaviours, non-judgemental atmosphere]

- What are your thoughts on this approach? – What do you like? What would you change? How would you normally start conversation?

[Show list of toothbrushing barriers with explanation as to where they came from]

- What do you think of the barriers to toothbrushing selected?
  - Are there any missing?
  - Do these barriers match with your experience working with parents?

[Introduce/show cards and how they are used - original Uitblinkers cards and alternatives – with photograph/illustration]

- - What are your thoughts on presenting the barriers like this?
  - What do you think of the cards themselves?
    - Design, pictures, size, number, ease of use
  - Which style of card do you prefer? Any ideas as to how they could be improved?
  - How useful to you think use of these cards would be in discussing toothbrushing with parents referred to the DHSW service?
  - How would parents respond to their use?
  - Do you currently use any materials that you bring on home visits e.g. leaflets, folders, tablets etc

[Introduce techniques – use case studies as examples]

- What about the tips/ strategies to help parents/ carers?
  - Do these ‘solutions’ match with your experience working with parents?
  - Would the suggestions for follow up work?
  - Do you currently leave any materials with parents e.g. reminder cards, information cards? Is this useful?
  - What do you think would be best to leave with families in this situation? ?reminder card for dentist appointment?
- What about your own ability to carry this out? Or your colleagues?
  - Confidence, skills, how it would feel
- What about parent / family factors?
  - Attitudes, acceptance, ability to understand, adherence?
  - How would you feel about carrying out this intervention?
- What are the practical issues with carrying out the intervention using these resources and/or what would help?
  - Space, time, home setting/environment/ other resources required
  - Management of the programme e.g. your job/ role/ training
  - We are building up some scenarios/case studies such as previously mentioned – would these be useful for training purposes?
- Which parents do you think this intervention would be most useful for?
  - Targeted? How to decide which parents?
- How do you think this fits with general initiatives/ policy for Scotland’s young people?
  - E.g. educational and other interventions, joined up working, community linking
- Is there anything else about the intervention we have not covered that you feel is important?
