# Supplementary material for: Developing a Prototype Home‐Based Toothbrushing Support Tool for Families in Scotland: A Mixed‐Methods Study With Modified Delphi Survey and Semi‐Structured Interviews
Source: Community Dent Oral Epidemiol. 2025 Feb 12;53(3):296–306. doi: 10.1111/cdoe.13031 (PMC12064878; doi:10.1111/cdoe.13031)
Supplement: Supplementary file 5 — Data S5. [file CDOE-53-296-s002.docx]

**Supplementary file 5 Template Analysis detail showing themes, subthemes and illustrations**

| Theme 1 The barriers present an exhaustive set and are valid from staff experience with families | | |
| --- | --- | --- |
| Subtheme code | **Subtheme descriptions** | **Subtheme illustrations** |
| Validity of barriers | The presented set match the barriers encountered in staff experience with families | Every one of them yeah, I’ve come across every one of them yeah. And I would say yeah, that would be the top of the list of barriers, definitely.  [Participant 2, Glasgow, 15 years experience]  I don’t think there’s anything within there that didn’t seem to fit in with what could potentially be causes of parents not […] sort of following through with advice given” [Participant 3, Glasgow, 14 years experience]  I think all very true. All of them. We’ve touched on most of them, really, just chatting, yes you know what the barriers are. We know what they are and just, I suppose it’s tailoring it for each family because every family’s individual, the child’s individual as well, isn’t it?” [Participant 7, Highland, 10 years experience] |
| Completeness of barrier set | The presented set cover most barriers encountered in staff experience with families | […] the list was good, really.  I mean, as you were reading them out, I’m like, oh, right, tick that one off in my head, then, you know. […] Yes, you did, yes, you got it all there. [Participant 6, Highland, 13 years experience]  I: and is there anything that you can think is missing from that list?  Off hand, I don't think [anything missing] so because again, you've got the finances. You've got lack of time […] I know, a family I had, been years experience and years experience ago I was involved with them. They just had a chaotic lifestyle that you know they all meant well, but it was just, the routine […] But I don't think there's anything missing there. [Participant 8, Highland 7, years experience]  Yeah, I think definitely we would face or our families would definitely face a good majority of those like we would come across that very often. And there be a lot of them that would be... Yeah, definitely. I think it’s broad enough that it would cover mostly everything. [Participant 9, Ayrshire and Arran, 10 years experience] |
| Theme 2 Motivational Interviewing is appropriate and fits with usual practice | | |
| Building rapport through positive opening | Opening the conversation with positive aspects helps build rapport | I think this is the right way to start a conversation - you want to build up a rapport and establish as positive conservation at the start [Participant 1, Glasgow, 15 years experience]  I like that starting off with what’s going well with it […] it’s something that the parents are going to feel good about that they can mention, even if it’s only one thing” [Participant 3, Glasgow, 14 years experience] |
| Current practice alignment | The overall motivational interviewing approach fits with current support worker practice | I think what you’re explaining’s generally how you would conduct your visit anyway, and you’d be looking for them to lead the conversation and kind of keeping them on track in terms of what the end goal was [Participant 4, Glasgow, 8 years experience]  I think that’s something that we kind of try to do anyway. To say, how are you getting on with the toothbrushing. Kind of try and engage them. Like you say you don’t want to go in with saying you’re doing everything wrong [Participant 4, Tayside, 12 years experience]  I think that’s the same kind of style that I’ve got anyway. You can’t be judgemental at all, you don’t know what some of these issues are and barriers and no I think it sounds quite positive. [Participant 10, Ayrshire and Arran, 8 years experience] |
| Theme 3 The included behaviour change techniques are workable | | |
| Use of cards as a prompt | Parent selected cards as a way to begin discussion on overcoming barriers | I think it’s a good prompt you know. […] because it might be the case where you know, although they’ve not got a tooth brushing routine in place, they might not have organised their mind enough to know why there’s not. Whereas something like that would maybe prompt them to say well that is the reason why […] So you know, the cards would probably prompt that like. Yeah, I think it’s a good idea. [Participant 2, Glasgow, 15 years experience]  I really like it because, although we would be there to guide and support, but I really like the idea that it’s actually the parent, themselves, that are coming up with what’s going to suit them, or what they want to try […] [Participant 3, Glasgow, 14 years experience] |
| Social reassurance | Cards can reassure parents they are not alone and they are facing socially normative issues | I think it would be good for parents who are certainly, have a barrier that are struggling to engage with you, you know, and you could say […] this has been proven that this seems to be a barrier for a lot of people […] especially if it's her first, they don't want to admit that they're struggling. So I think for parents to see other people have these barriers and they find that they’re struggling with this side of it, it’s probably good. [Participant 9, Ayrshire, 10 years experience] |
| Behaviour  change tips and strategies | Tips on the cards as a way to approach barriers | I think [the tips and strategies] they're good. I mean, I think anything or even to try anything new or different, is always going to be good. Because sometimes you know parents. I mean, I'm the same, you know, with anything you know, somebody will suggest something. I think well I never thought that you know. And just doing it in a different way. [Participant 8, Highland, 7 years experience]  I found them really helpful. I would imagine any parent would as well you know, especially if they’re struggling. And most of the time parents will take advice and suggestions, maybe not so much advice but suggestions to say look, you say it and if you bite off a small chunk, that’s manageable [Participant 2, Glasgow, 14 years experience] |
| Visual nature of cards | Pictorial/ visual cards viewed as helping the conversation | Aye, fine, absolutely fine. […] Anything visual for me helps, anything visual. […] And it's always, like when you talk to people you get into it, and the person is like that, and there's other stuff going on. So, no, anything visually is a good thing. [Participant 5, Glasgow, 4 years experience] |
| Follow-up visits and materials | Perceived need for follow up visits and using materials to supplement behaviour change techniques | Because sometimes they’re listening to you, but maybe not taking everything in, you know, but then when you leave something […] so, yes, I think it would be good if there is, you know [...]something, yes, the techniques that we’re asking them, you know, to do […] and maybe a tick-sheet. [Participant 2, Glasgow, 14 years experience]  It would work because I mean I do, I do try and you know, follow up. You know every maybe three or four months just to see how things are. [Participant 8, Highland, 7 years experience]  Maybe that's something [reminders] they could put on the fridge or something that could look at to, you know with different tips to try or different techniques to try. [Participant 12, Tayside, 10 years experience] |
| Theme 4 The tool is generally feasible within the operation of Childsmile home visits | | |
| General feasibility | Proposed tool generally felt to be feasible for home delivery | Aye, they're really good, I think they're really good. And they would probably work during a home visit as well. [Participant 5, Glasgow, 4 years experience] |
|  |  |  |
| Physical tool preference | Preference for paper/ physical tool over electronic delivery | I would rather have pictures because sometimes, you know, technology can [not] work […] when I do my visits we could take a tablet and just type while we’re talking, but I feel that I’m not giving them the full attention. I would prefer cards. [Participant 1, Glasgow, 15 years experience]  I think cards are probably better. […] the kids, toddlers, they're gonna come. They're going to touch everything you’ve got, like they're physically going through your stuff. And if you've got a tablet, chances are they're going to know their way around it. And they’ll probably want to be looking for apps or YouTube or something [Participant 9, Ayrshire, 10 years experience] |
| Portability of tool | Smaller size cards indicated for practical reasons, laminated and clipped together | I do think they're good. Do you think the size is a wee bit kind of large, [name]? Would these be, like clipped together in some kind of little ring-binder or something? […] I think there needs to be an option of clipping them in, so that they don't get lost, or whatever, and laminated even, so that they're kept in good nick, if you know what I mean. Because it's so easy, if you're taking these out two, three, four times a day, eventually, especially if there's toddlers in the house, they come over and grab it or whatever.  [Participant 3, Glasgow, 14 years experience]  [Participant gets A5 work folder]: […] it’s got everything in it so I don’t know if the cards could maybe be added to this? I think that’s quite a good call because this…we call it the bible so it’s got everything, you know. [Participant 6, Highland, 13 years experience]  I love visual aids I’ve got lots of them so I’m sure 11 cards could fit in my bag quite nicely. But some people just go with a toothbrush and toothpaste. Weve all got our own different styles. Some people might not like them, but I think I could fit them in my bag somewhere and use them.  [Participant 10, Ayrshire, 8 years experience] |
| Theme 5 Children with additional support needs require a tailored approach | | |
| Longer term resistance | Children with special needs take longer to overcome barriers | What I find particularly difficult is there seems to be quite a few families now where their child is on the autistic spectrum […] ordinarily you would maybe get a child who refuses to brush their teeth but you know, that’s transitory they’ll eventually come out. Whereas autism is an ongoing thing [Participant 2, Glasgow, 15 years experience] |
| Sensory barriers | Some children resist toothbrushing due to texture and/or flavour | […] what we’re starting to find a lot more just now is the amount of specialist dental services we need, so for like autistic kids, and stuff like that, it seems to be a lot higher than what it ever was before. [..,] They're the type of kids where it's really difficult to brush their teeth for mums and dads. […] I had a mum a couple of weeks ago who phoned me and was at her wits end. She's been great, she keeps an eye on his diet, really healthy stuff, but cannot get a toothbrush in his mouth, he just hates the texture. She's tried it with a finger brush, tried different things, doesn’t like anything. [Participant 5, Glasgow, 4 years experience]  A lot of parents who have got autistic children who don’t like the texture of the toothbrush or the flavours of the toothpaste [Participant 4, Glasgow, 8 years experience]  And my experience even with the families that I have tried to support a wee bit further […] often the child is maybe later on diagnosed with ADH or autism, you know, sort of thing. And there’s maybe been sensory issues. I always ask that. Do they think there’s any sensory things going on regards touch, taste, you know, when I get referrals? [Participant 7, Highland, 10 years experience] |
